# Functioning of the Photosynthetic Apparatus in Response to Drought Stress in Oat × Maize Addition Lines

**DOI:** 10.3390/ijms21186958

**Published:** 2020-09-22

**Authors:** Katarzyna Juzoń, Dominika Idziak-Helmcke, Magdalena Rojek-Jelonek, Tomasz Warzecha, Marzena Warchoł, Ilona Czyczyło-Mysza, Kinga Dziurka, Edyta Skrzypek

**Affiliations:** 1 Department of Biotechnology, The *Franciszek Górski* Institute of Plant Physiology, Polish Academy of Sciences, Niezapominajek 21, 30-239 Krakow, Poland; m.warchol@ifr-pan.edu.pl (M.W.); i.czyczylo@ifr-pan.edu.pl (I.C.-M.); k.dziurka@ifr-pan.edu.pl (K.D.); e.skrzypek@ifr-pan.edu.pl (E.S.); 2 Institute of Biology, Biotechnology, and Environmental Sciences, University of Silesia in Katowice, Jagiellońska 28, 40-032 Katowice, Poland; dominika.helmcke@us.edu.pl (D.I.-H.); magdalena.rojek@us.edu.pl (M.R.-J.); 3 Department of Plant Breeding, Physiology, and Seed Science, University of Agriculture in Krakow, Podlużna 3, 30-239 Krakow, Poland; tomasz.warzecha@urk.edu.pl

**Keywords:** *Avena sativa* L., chlorophyll *a* fluorescence, drought, GISH, OMA line, photosynthetic apparatus, *Zea mays* L.

## Abstract

The oat × maize chromosome addition (OMA) lines, as hybrids between C3 and C4 plants, can potentially help us understand the process of C4 photosynthesis. However, photosynthesis is often affected by adverse environmental conditions, including drought stress. Therefore, to assess the functioning of the photosynthetic apparatus in OMA lines under drought stress, the chlorophyll content and chlorophyll *a* fluorescence (CF) parameters were investigated. With optimal hydration, most of the tested OMA lines, compared to oat cv. Bingo, showed higher pigment content, and some of them were characterized by increased values of selected CF parameters. Although 14 days of drought caused a decrease of chlorophylls and carotenoids, only slight changes in CF parameters were observed, which can indicate proper photosynthetic efficiency in most of examined OMA lines compared to oat cv. Bingo. The obtained data revealed that expected changes in hybrid functioning depend more on the specific maize chromosome and its interaction with the oat genome rather than the number of retained chromosomes. OMA lines not only constitute a powerful tool for maize genomics but also are a source of valuable variation in plant breeding, and can help us to understand plant susceptibility to drought. Our research confirms more efficient functioning of hybrid photosynthetic apparatus than oat cv. Bingo, therefore contributes to raising new questions in the fields of plant physiology and biochemistry. Due to the fact that the oat genome is not fully sequenced yet, the mechanism of enhanced photosynthetic efficiency in OMA lines requires further research.

## 1. Introduction

Oat (*Avena sativa* L. 2n = 6x = 42) and maize (*Zea mays* L. 2n = 2x = 20) are the most remotely related plant species of which we are aware that can be sexually hybridized and produce stable fertile partial hybrids [1]. The discovery of the presence of maize chromosomes in oat plants after crossing with maize and obtaining oat lines with added maize chromosomes [2], called OMA (oat × maize chromosome addition) lines, initiated their use in order to simplify analysis of the maize genome [1]. Many studies have been performed so far employing OMAs, including analyses of maize knob and centromere structure [2], meiotic chromosome behavior [3], physical mapping of single-copy sequences on maize chromosomes by fluorescence in situ hybridization (FISH) [4], and flow-cytometry sorting of an individual maize chromosome [5]. Other applications are studies on the expression of maize genes in the genetic background of oat covering analyzing gene regulation aspects [6] and possible acquisition of new features [7] and/or disease resistance [8]. The OMA lines, as hybrids between C3 and C4 plants, can help to explore the process of C4 photosynthesis [7]. OMA lines may find use in the study of the genetics of the C4 pathway of maize and identify chromosomes/chromosome regions important in this process [7,9,10]. These lines enable elimination of undesired maize chromatin and stabilize transmission of the wanted ones. According to Tolley et al. (2012) some OMA lines change leaf anatomy, e.g., reduced vein spacing, which is connected with C4 photosynthesis; however, this was not as low as in maize. That vein spacing can be altered in a C3 leaf but appears to be dependent on the genomic context. It is possible that the *loci* on maize chromosome 9 encoding this trait are differentially silenced depending on their interaction with the oat genome or that they interact differently with specific oat alleles. Their study showed that C4 plants also had larger parenchymal bundle sheath cells that contain more chloroplasts than those of C3. OMA lines possessing maize chromosome 1, 2, 3, 7, 8 and 9 had significantly larger cell areas than the oat parental lines. Moreover, the presence of maize chromosome(s) often leads to morphological (e.g., thickened shoots, straight leaf blade, bent panicle) and physiological (e.g., disturbed panicle development and chlorophyll synthesis) abnormalities, the nature of which depends on the specific maize chromosome and oat genotype [9]. Therefore, their impact on the functioning of the photosynthetic apparatus is also postulated, as well as the increased tolerance of the OMA lines to environmental factors, e.g., corona rust [11]. 

However, photosynthesis efficiency can be adversely affected by the environmental conditions which lead to functional and structural changes in the photosynthetic apparatus [12], and in turn limit plant growth and productivity [13,14]. Among abiotic stresses, drought is considered as one of the most important factors limiting the productivity of crops, e.g., rice, wheat, and maize [15]. Severe drought also decreases chlorophyll content in leaves and causes damage in the photosynthetic apparatus, mainly in PSI and PSII [16]. However, the response of the photosynthetic linear electron transport process, particularly the interaction between two photosystems under stress conditions, remains still unclear [17]. Chlorophyll is unarguably the key component enabling vegetation photosynthesis, and therefore is an important indicator of plant function and productivity [18]. In unfavorable environmental conditions, plants have developed remarkable abilities to modulate growth and development mechanisms through changes at the physiological, biochemical and genetic levels. The sensitivity of plants to drought depends on stress intensity, species, genotype, the presence of other stress factors, and the developmental phase of plant [19].

This experiment was performed in order to estimate variation in functioning of the photosynthetic apparatus among diverse oat × maize chromosome addition lines with different numbers of preserved maize chromosome(s). In the present study we analyzed the chlorophyll content and chlorophyll *a* fluorescence parameters in OMA lines under drought conditions. We hypothesized that differences in photosynthetic performance under drought conditions will be displayed by individual OMA lines, since there are also considerable genetic and physiological differences between them. To our knowledge, this is the first study combining photosynthetic efficiency in OMA lines under drought stress.

## 2. Results

### 2.1. Detection of OMA Lines

**Figure 1:**
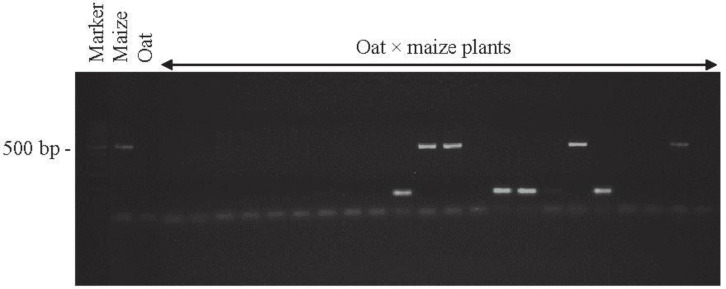
Identification of oat × maize F1 plants. PCR products of genomic DNA of oat, maize, and a selection of 22 oat × maize F1 plants shown after electrophoresis in 1.5% (*w*/*v*) agarose gel. Bands represent 500 bp DNA fragments that were amplified with marker *Grande-1*. Marker leader is shown in the first line. Maize cv. Waza specificity is shown by product presence in maize DNA (positive control) and absence in oat cv. Bingo DNA (negative control). The presence of retained maize chromosomes is indicated in 4 of the F1 plant DNAs shown.

OMA lines obtained by crossing oat with maize were identified by the PCR product (500 bp) amplified with primers specific for the maize retrotransposon *Grande-1* (GenBank Accession No. X97604; [2]). Copies of the *Grande-1* are widely distributed on each maize chromosome. Figure 1 shows an agarose gel with 500 bp product typical for the maize retrotransposon *Grande-1*. Despite the expected products we also obtained some additional amplicons. While the bands are quite strong, and located on the level of 100 bp of molecular marker weight, they seemed to be rather nonspecific amplification products rather than primer-dimer, which also appears at the bottom line.

**Figure 2:**
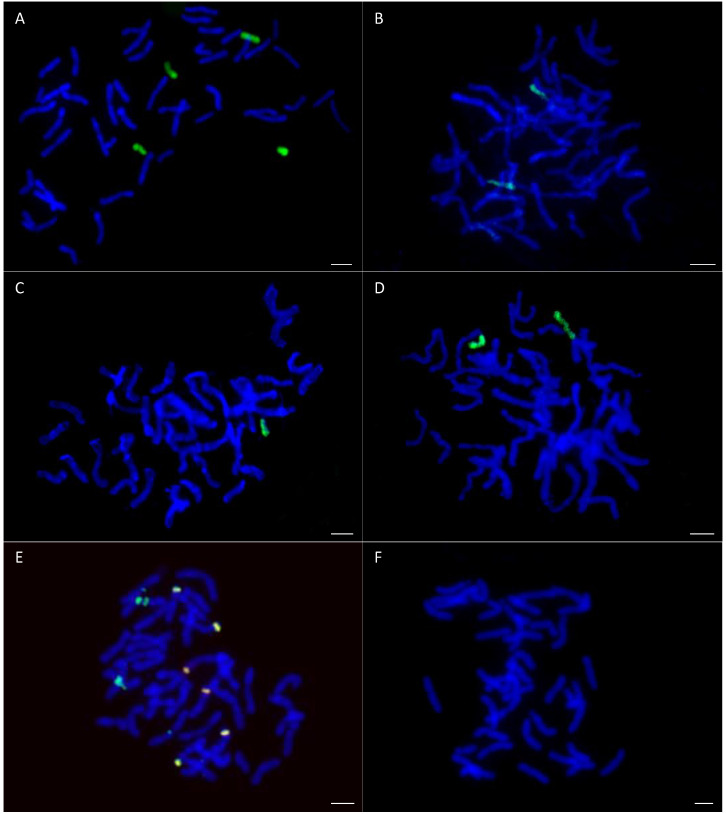
Visualization of added maize chromatin in oat genome by genomic in situ hybridization (GISH): (**A**) line I, (**B**) line II, (**C**) line III, (**D**) line IV, (**E**) line V, (**F**) cv. Bingo. Green fluorescence: maize total genomic DNA (gDNA); red fluorescence: 25S rDNA; blue fluorescence: DAPI. Yellow fluorescence is the result of colocalization of red and green signals and indicates the presence of highly conservative 35S rDNA sites, which are recognized by both maize gDNA and 25S rDNA probe. Scale bar: 5 µm.

Genomic in situ hybridization (GISH) with maize gDNA as a probe was performed on OMA lines in order to verify the number of added chromosomes (Figure 2A–E). Oat cv. Bingo was used as a control possessing a clear set of *Avena sativa* L. chromosomes (Figure 2F). All of the analyzed OMA lines had a full set of oat chromosomes.

The number of detected maize chromosomes differed between the OMA lines (Table 1). Identification of added maize chromosomes was done by simple sequence repeat (SSR) markers. The OMA line I was a double disomic characterized by the presence of maize chromosomes 1 and 2 (2*n* = 46 (42 + Zm1″ + Zm2″)) (Figure 2A). Line II had a pair of chromosome 2 (2 *n* = 44 (42 + Zm2″)) (Figure 2B). Line III comprised chromosome 3 of maize genome (2 *n* = 43 (42 + Zm3′)) (Figure 2C). Line IV had two chromosome 5 (2 *n* = 44 (42 + Zm5″)) (Figure 2D) and line V had chromatin fragments of chromosome 6 (Figure 2E).

### 2.2. Comparison of OMA Lines in Control Conditions

The functioning of the photosynthetic apparatus was assessed on the basis of the total chlorophyll content (SPAD), the content of photosynthetic pigments (chlorophyll *a*, *b*, carotenoids) and selected chlorophyll *a* fluorescence parameters.

The analysis of variance showed significant differences between tested oat plants with optimal water supply in terms of the content of total chlorophyll and photosynthetic pigments, as well as most of the chlorophyll *a* fluorescence parameters (Table 2). There were no significant differences among oat plants in the case of maximum photochemical efficiency of PSII (F_v_/F_m_) and energy dissipated from PSII (DI_0_/CS).

**Figure 3:**
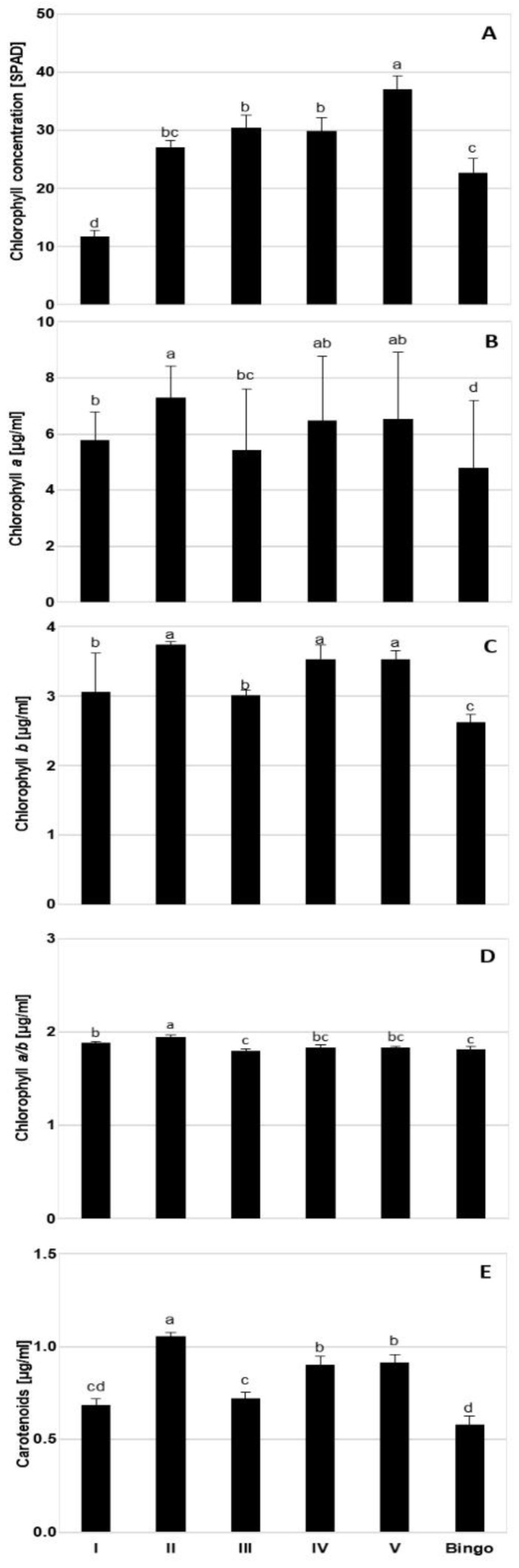
The content of photosynthetic pigments in the leaves of OMA (oat × maize chromosome addition) lines and cv. Bingo in control condition (70% field water capacity, FWC): (**A**) chlorophyll concentration [SPAD unit]; (**B**) chlorophyll *a* [µg/mL]; (**C**) chlorophyll *b* [µg/mL]; (**D**) chlorophyll *a/b* [µg/mL]; (**E**) carotenoids [µg/mL]. The mean values (n = 5) ± SE marked with different letters are significantly different at *p* ≤ 0.05 according to the Duncan’s multiple test.

The chlorophyll concentration in SPAD units showed differences between tested lines (Figure 3). The highest values were obtained by line V, and the lowest by line I. However, the content of photosynthetic pigments showed different value distributions: tested OMA lines showed higher pigment content compared to cv. Bingo, which was characterized by the lowest content of chlorophyll *a* (Chl*_a_*), chlorophyll *b* (Chl*_b_*), and carotenoids (Car). Among examined OMA lines, three of them had greater pigment content: line II, IV (with two preserved maize chromosomes) and V (with preserved maize chromatin). Line I (with four preserved maize chromosomes) showed intermediate values between cv. Bingo and other OMAs.

**Figure 4:**
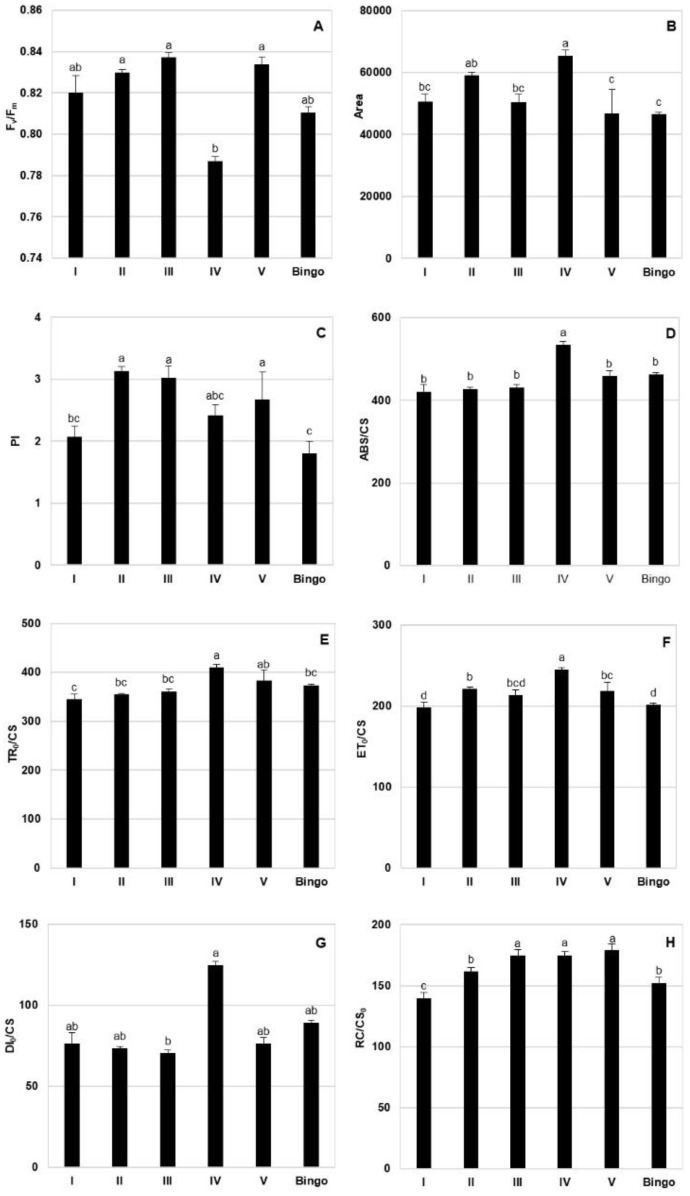
Chlorophyll *a* fluorescence parameters in the leaves of OMA lines and cv. Bingo in control condition (70% FWC). (**A**) F_v_/F_m_—maximum photochemical efficiency of PSII; (**B**) Area—area over the chlorophyll *a* fluorescence induction curve; (**C**) PI—overall performance index of PSII photochemistry; (**D**) ABS/CS—light energy absorption; (**E**) TR_0_/CS—excitation energy trapped in PSII reaction centers; (**F**) ET_0_/CS—energy used for electron transport; (**G**) DI_0_/CS—energy dissipated from PSII; (**H**) RC/CS_0_—number of active reaction centers in the excited leaf fragment. The mean values (n = 5) ± SE marked with different letters are significantly different at *p* ≤ 0.05 according to the Duncan’s multiple test.

To assess the functioning of the photosynthetic apparatus, some parameters of chlorophyll *a* fluorescence were selected (Figure 4). The presented data showed that the line IV usually achieved the highest CF parameters apart from F_v_/F_m_ (Figure 4A) and PI (Figure 4C) compared to the other OMA lines as well as cv. Bingo. Plants of line IV were characterized by greater area over the chlorophyll *a* fluorescence induction curve (Area) (Figure 4B), which corresponds to the size of the reduced plastoquinone pool. Photosynthetic apparatus of line IV showed higher light energy absorption (ABS/CS) (Figure 4D), as well as excitation energy trapped in PSII reaction centers (TR_0_/CS) (Figure 4E). These plants used more energy for electron transport (ET_0_/CS) (Figure 4F) while showing higher energy dissipation from PSII (DI_0_/CS) (Figure 4G), although they were not the only ones with more active reaction centers (RC/CS_0_) (Figure 4H). Line I and cv. Bingo were characterized by lower CF parameter values, similarly observed with regard to the pigment content. 

### 2.3. Assessment of OMA Lines in Drought Condition

The analysis of variance showed significant differences in the relative water content in leaves (LRWC) only in terms of applied treatment (control or drought), regardless of plant genotype (Table 3). However, the application of treatment did not significantly affect the content of Chl*_b_* and Car, while term of measurements had no impact on Chl*_a/b_* ratio. The interaction of the genotype with the treatment turned out to be significant only in Chl*_a/b_* ratio. The interaction of treatment with term and the interaction of genotype with treatment and with term were significant for all pigments.

**Figure 5:**
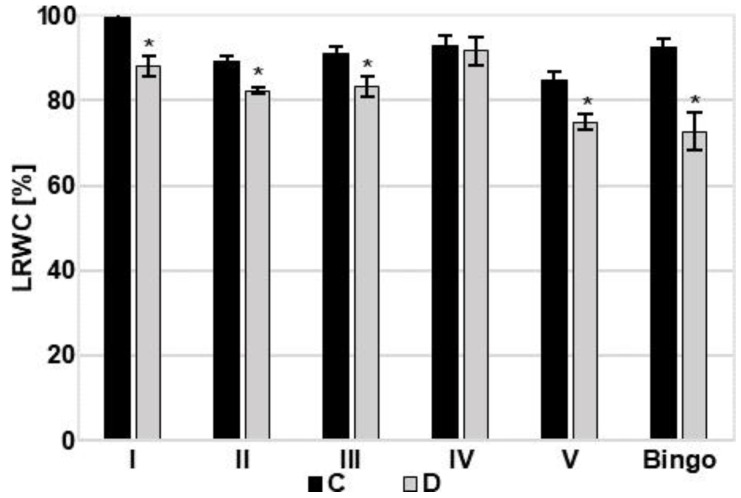
Leaf relative water content (LRWC) [%] of OMA lines and cv. Bingo on the 14th day of drought. C—control, 70% FWC, black bars; D—drought, 25% FWC, grey bars. The mean values (n = 5) ± SE marked with an asterisk (*) are significantly different at *p* ≤ 0.05 according to the Student’s t test.

To verify the impact of drought stress level on tested plants, the leaf relative water content (LRWC) was measured. The applied 14-day drought caused a decrease in the LRWC value in most of the tested lines as well as cv. Bingo (Figure 5). Compared to control plants, the greatest decline of LRWC by 21% was recorded in cv. Bingo. In drought condition, lines I and V showed reduction of LRWC by approx. 11%, while lines 23 and 55 reduced by approx. 7–8%. There were no significant changes in LRWC values in line IV.

**Figure 6:**
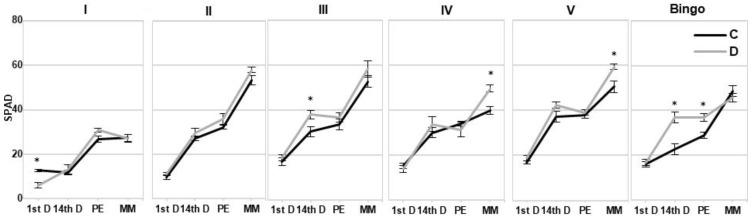
The chlorophyll concentration in SPAD units in the leaves of OMA lines and cv. Bingo on the 1st day of drought (1stD), 14th day of drought (14thD), panicle ejection phase (PE), kernel milk maturity phase (MM). C—control, 70% FWC, black line; D—drought, 25% FWC, grey line. The mean values (n = 5) ± SE marked with an asterisk (*) are significantly different at *p* ≤ 0.05 according to the Student’s t test.

The chlorophyll concentration in SPAD units did not show significant differences in the majority of the tested oat lines regardless of the plant developmental phase in which the SPAD readings were taken (Figure 6). However, an increase in SPAD values was observed during plant development. Few statistically significant differences were noted in line I on 1st day of drought, lines III and cv. Bingo on 14th day of drought, cv. Bingo in panicle ejection phase and lines IV and V in the kernel milk maturity phase. In most cases, plants subjected to drought stress had higher SPAD values by about 20% compared to control conditions, with cv. Bingo by about 38%. Only line I on the 1st day of drought showed more than 50% decrease of SPAD compared to control plants.

**Figure 7:**
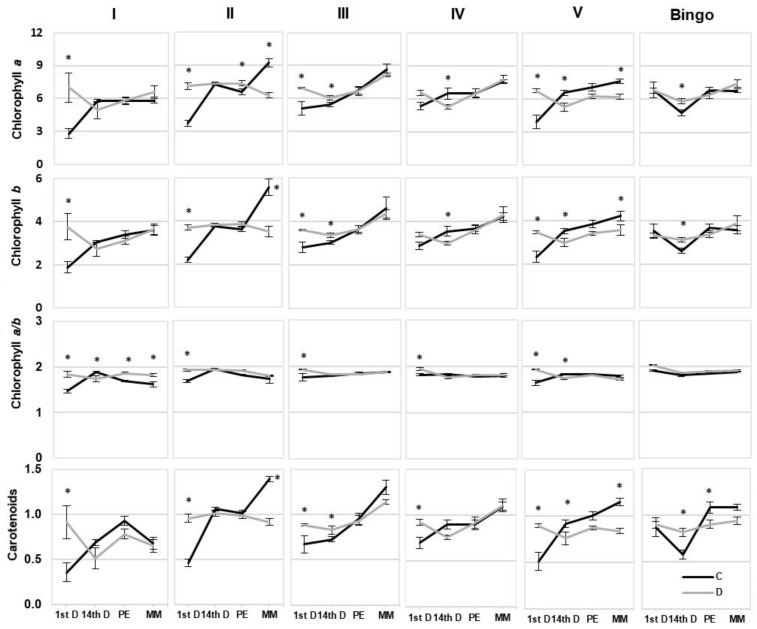
The content of photosynthetic pigments [µg/mL] in the leaves of OMA lines and cv. Bingo on the 1st day of drought (1stD), 14th day of drought (14thD), panicle ejection phase (PE), and kernel milk maturity phase (MM). C—control, 70% FWC, black line; D—drought, 25% FWC, grey line. The mean values (n = 5) ± SE marked with an asterisk (*) are significantly different at *p* ≤ 0.05 according to the Student’s t test.

The content of photosynthetic pigments (Figure 7) did not show such a clear change depending on the plant’s developmental phase such as the SPAD measurements (Figure 6). It seemed that applied drought stress had the strongest effect on the content of chlorophylls (Chl) and carotenoids (Car) on 1st day of drought, and most of tested lines recorded a significant increase in their content compared to control conditions. The highest increase in Chl*_a_* and Chl*_b_* content was observed in lines I and II by approx. 60% and 50%, respectively, and in line V by approx. 40%. Similar trends were shown in the content of Car., whereas 14-day drought treatment caused a decline by 20% in the Chl content in the leaves of two tested lines IV and V, which was maintained during further plant growth only in line V. In turn, in line II a decrease in the content of photosynthetic pigments was noted only in the kernel milk maturity phase and it declined by about 47% of Chl*_a_*, 58% of Chl*_b_*, and 35% of Car. In this phase, a significant decrease in the above-mentioned pigments was also observed in line V and it oscillated around 20% compared to the control conditions.

The analysis of variance for chlorophyll *a* fluorescence parameters showed significant differences between tested oat plants under drought conditions in terms of both single traits and their interaction (Table 4). Almost all traits/interactions were significant at *p* ≤ 0.001.

**Figure 8:**
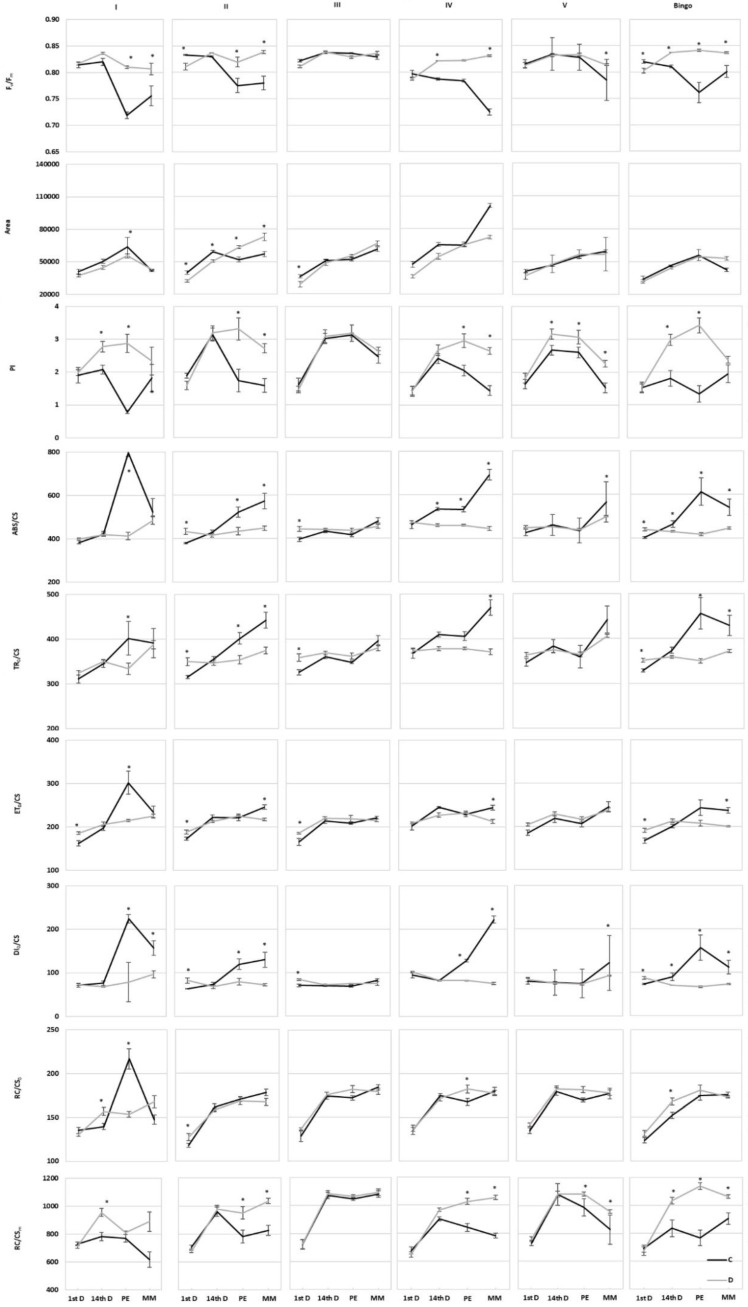
Chlorophyll *a* fluorescence parameters in oat leaves on 1st (1stD) and 14th (14thD) day of drought, panicle ejection (PE) and milk maturity (MM) phase. F_v_/F_m_—maximum photochemical efficiency of PSII; Area—area over the chlorophyll a fluorescence induction curve; PI—overall performance index of PSII photochemistry; ABS/CS—light energy absorption; TR_0_/CS—excitation energy trapped in PSII reaction centers; ET_0_/CS—energy used for electron transport; DI_0_/CS—energy dissipated from PSII; RC/CS_0_—number of active reaction centers in the excited leaf fragment; RC/CS_m_—number of active reaction centers in the relaxation state of leaf fragment; C—control, 70% FWC, black line; D—drought, 25% FWC, grey line. The mean values (n = 5) ± SE marked with an asterisk (*) are significantly different at *p* ≤ 0.05 according to the Student’s t test.

The values of chlorophyll fluorescence parameters during plant development were characterized by some variation between the tested lines. An increase in the F_v_/F_m_ and PI was observed in the leaves of most of the examined OMA lines and cv. Bingo subjected to drought (Figure 8). Furthermore, the Area parameter showed an increase in line II in the panicle ejection and kernel milk maturity phase, indicating an increase in the size of the acceptor pool in PSII. Indicators allowing the estimation of energy flow calculated on the excited surface of the photosynthetically active sample (CS) were characterized by a similar response in control and stress condition, which may indicate proper photosynthetic efficiency. In most of plants subjected to drought, a decrease in the flow of photon flux absorbed by chlorophyll molecules of PSII antennas (ABS/CS) and retained in a single PSII reaction center (TR_0_/CS) was observed, as well as a decrease in thermal energy dissipation in the PSII reaction centers (DI_0_/CS). However, drought had less of an impact on the electron transport through PSII (ET_0_/CS), which may indicate a certain adaptability of the tested lines to adverse conditions. In addition, in most cases there were no significant changes in the number of active reaction centers in the excited leaf fragment (RC/CS_0_), while their amount increased in the relaxed state (RC/CS_m_). The only exception from this rule was line I in the panicle ejection phase.

**Figure 9:**
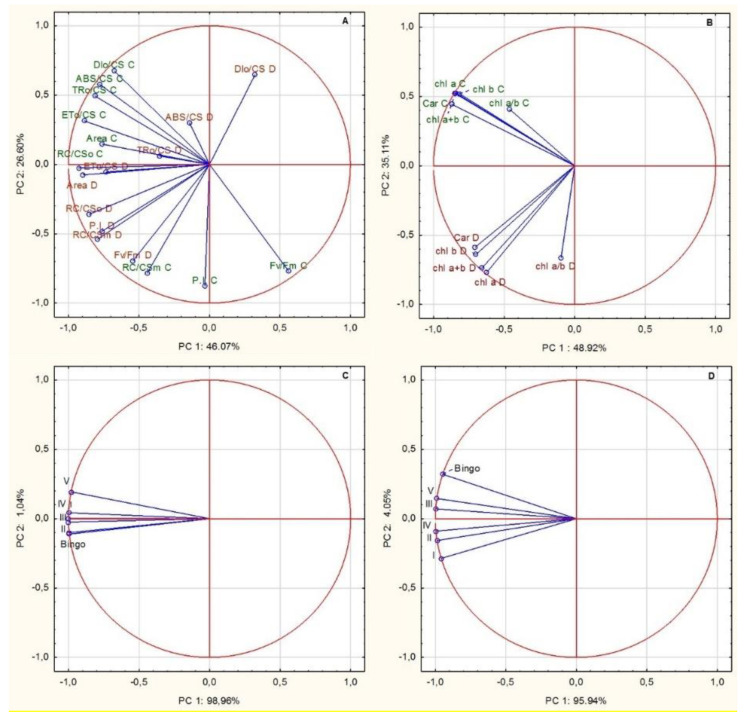
Biplot based on the first two principal component axes (PC 1 and PC 2); chlorophyll *a* fluorescence parameters (**A**) and photosynthetic pigments (**B**) dependent on the treatment; distribution of cv. Bingo and OMA lines based on the chlorophyll *a* fluorescence parameter (**C**,**D**) photosynthetic pigments.

The principal components analysis (PCA) indicated that the measured chlorophyll *a* fluorescence parameters could distinguish the treatment of the plants as they are all together compared for all the parameters. Acute angle flanked by the parameters means positive correlation, whereas an obtuse angle is negative, and right angle means no correlation. Both PCs (principal components) accounted for 72.67% of the total variation of the data set for chlorophyll *a* fluorescence parameters and 84.03% for photosynthetic pigments (Figure 9A,B). PCA revealed that the first PC explained 46.07% of the variation with chlorophyll *a* fluorescence parameters (Figure 9A) and 48.92% with photosynthetic pigments (Figure 9B). The second PC explained 26.60% and 35.11% of the total variability with chlorophyll *a* fluorescence parameters (Figure 9A) and photosynthetic pigments (Figure 9B), respectively. These parameters are able to separate plants in association with drought treatment.

PCA also indicated that chlorophyll *a* fluorescence parameters and photosynthetic pigments grouped tightly examined hybrids and cv. Bingo (Figure 9A,C,D). PCA showed that the first PC explained 98.96% of the variation with chlorophyll fluorescence (Figure 9C) and 95.94% with photosynthetic pigments (Figure 9D). 

### 2.4. Morphological Characteristics and Yield Formation 

The analysis of variance showed significant differences between tested oat plants under drought conditions in terms of selected yield components (Table 5). Treatment did not affect the seed weight and its interaction with genotype had no influence on shoots and seeds number.

The tested lines were differentiated in terms of plant height (the tallest were V and Bingo plants, and the smallest line IV), number of side tillers (the largest number was developed by the plants of line III and the smallest by line V) and the term of entering subsequent development phases (line III and the cv. Bingo were the first to begin to throw out panicles; data not shown). 

**Figure 10:**
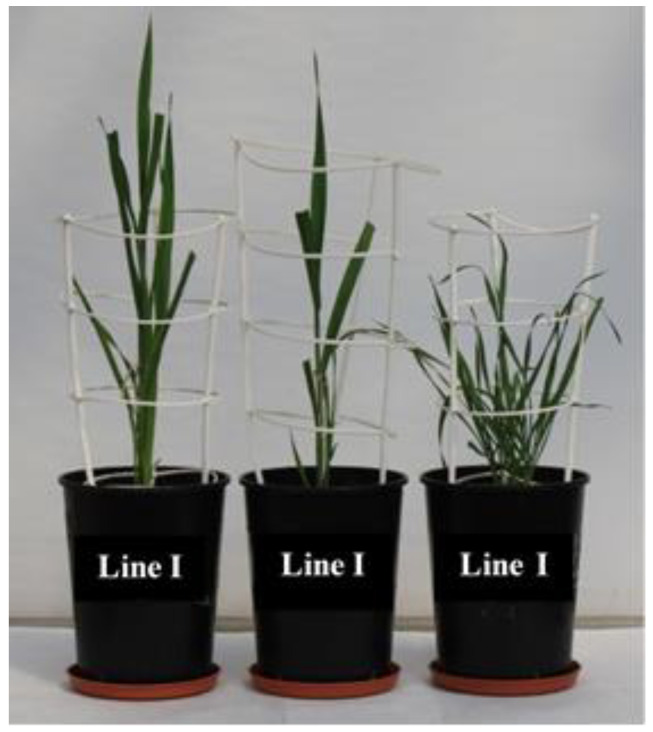
Morphological diversity of the hybrid line I.

**Figure 11:**
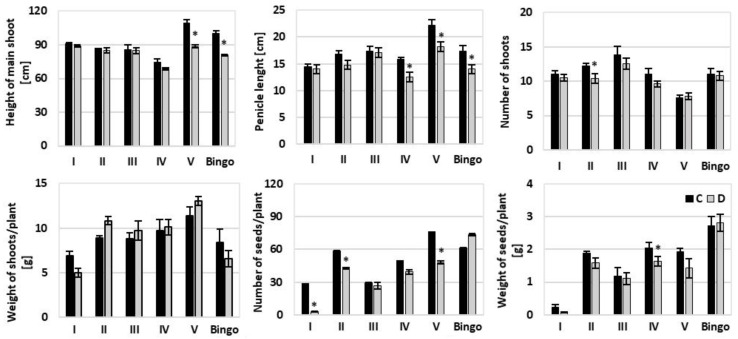
The impact of drought stress on the yield components of oat plants: height of the main shoot [cm], panicle length [cm], number of shoots, weight of shoots per plant [g], number of seeds per plant, weight of seeds per plant [g]. C—control, 70% FWC, black bars; D—drought, 25% FWC, grey bars. The mean values (n = 5) ± SE marked with an asterisk (*) are significantly different at *p* ≤ 0.05 according to the Student’s t test.

Among tested OMA lines only line I showed in a bushy type of growth, without a clearly formed main shoot (Figure 10). Moreover, the observed phenotypic differences only in some cases turned out to be statistically significant. Presented results suggested that applied drought had the strongest impact on the yield components of the line V and the cv. Bingo, where a decline of height of the main shoot and panicle length by approx. 19% was confirmed (Figure 11). Moreover, in line V subjected to drought, the number of seeds per plant decreased by 36% compared to control condition. In turn, line IV was characterized by a decline of two yield components: panicle length as well as weight of seeds by approx. 20%. Plants of line II under drought had fewer side shoots and seeds by 15% and 27%, respectively.

## 3. Discussion

In oat and maize crosses, some maize chromosomes are not extruded during embryogenesis, but are ultimately stabilized and act as oat chromosomes in mitosis [20]. Studies of Riera-Lizarazu et al. [21] explained that maize chromosome elimination in crosses between oat and maize is more gradual than, for example, in wheat and maize. A single maize chromosome in an oat genome background represents only about 2% of the total DNA [22]. Moreover, the OMA plants look like oat with some morphological modifications, and the amount of maize gene expression or gene silencing is difficult to intuitively assess. However, Dong et al. [10] actually found that more than 70% of the genes from the alien maize chromosomes maintained the original expression or transcription pattern under the oat genomic environment. They speculated that the maize genes maintaining the original transcription in OMA lines (introgression expression) may be predominantly regulated by a local *cis* element in the alien maize chromosome [10]. Gene expression of alien chromosomes has also been detected in wheat × barley addition lines [23]. 

### 3.1. Functioning of the OMA Lines Photosynthetic Apparatus in Optimal Hydration

Despite the OMA lines being valuable genetic materials, they are also used in the examination of expression of the C4 photosynthetic system [7,9,10]. The results presented in our study indicated some differences between tested OMA lines and *A. sativa* cv. Bingo. It is worth stressing that in the case of most of the analyzed CF parameters, OMA lines were characterized by higher values, which may confirm more efficient functioning of their photosynthetic apparatus. Their suggested predominance can be also reflected in the higher pigment content (Chl*_a_*, Chl*_b_*, Car) compared to oat cv. Bingo. Furthermore, results for OMA lines also exceed the values obtained for doubled haploids (DH) of oat [24]. The greatest difference was observed in the Area, and line IV showed almost two times higher values of this parameter. Among the examined OMA lines, line IV, with two retained maize chromosomes (2*n* = 44 (42 + Zm5″)), showed higher values of most CF parameters. Interestingly, line II, which also comprises two maize chromosomes (2*n* = 44 (42 + Zm2″)), was characterized by lower photosynthetic efficiency compared to line IV. Therefore, an important issue seems to be not only the number of retained chromosomes but also the kind of introduced maize chromosomes as well as oat genome. Moreover, line I with four retained maize chromosomes (2*n* = 46 (42 + Zm1″ + Zm2″)) turned out to be less efficient in the context of the functioning of the photosynthetic apparatus. In our opinion, these results can confirm that preserved maize chromosomes can positively affect the photosynthetic apparatus but this effect relies not only on the amount but also on the number of retained maize chromosome. Nevertheless, we are aware that a greater number of repetitions would provide even more reliable results and possibly reveal differences that were not statistically significant in the conducted experiment. Therefore, further studies including additional OMA lines are planned to gather more insightful information on the hybrids’ functioning. 

### 3.2. Functioning of the OMA Line Photosynthetic Apparatus in Drought Conditions

Drought is the most severe stress and the main cause of significant losses in growth and productivity of crop plants [25], inducing meaningful alterations in plant physiology and biochemistry. Various physiological responses of plants to drought are linked with their tolerance mechanisms such as pigment content and stability, as well as high relative water content [26]. The sensitivity of plants to drought depends on the intensity of stress, species, genotype, the presence of other stress factors, and the plant developmental phase [19]. 

Under drought stress, LRWC is proposed as a more important indicator of water status than other water potential parameters [27]. A significant decrease in LRWC value in response to drought stress has been reported by many authors [28,29,30,31]. Depending on the reduction of LRWC, water stress can be divided into mild (LRWC decreases by 8–10%), moderate (10–20%), and severe (over 20%) [32]. According to this classification, the soil drought applied in our experiment caused severe/moderate stress in cv. Bingo (LRWC decreased by 21%), and mild/moderate in tested OMA lines (LRWC declined by 7–11%). This deviation in LRWC may be attributed to differences in the ability of water absorption from the soil and/or controlling water loss through the stomata. It may also be caused by differentiated accumulation and osmotic adjustment to maintain tissue turgor and hence physiological activities [27]. Soluble sugars and proline are well known as the two most important organic solutes and their increase is commonly observed in response to environmental stress. Many studies indicate that evaluation of proline accumulation can be useful in the assessment of drought tolerance in cereals [33].

Despite the observed reduction in LRWC values we did not note such a clear response in the chlorophyll content under drought conditions. Chl degradation is listed as one of the consequences of drought stress that may result from sustained photoinhibition and photo-bleaching [34], even though other plant processes, such as cell division and cell expansion, are the earliest to respond to water deficit stress [35]. According to O’Neill et al. [36] a decline in SPAD index is a sensitive and readily measurable trait that could be used to screen for stress tolerance. Cassol et al. [37] showed that the SPAD index was linear and positively correlated with the Chl content, despite the fact that the reading of the instrument cannot be regarded as the absolute content of the Chl in the leaves. However, our results did not confirm these conclusions. Moreover, presented studies showed a significant discrepancy between SPAD index and chlorophyll content, particularly in lines IV and V, at kernel milk maturity phase—an increase of SPAD values was not reflected in the content of Chl measured spectrophotometrically. Therefore, it seems that the SPAD should not be treated as an independent indicator of tolerance, especially under mild drought stress, which was also observed in our earlier studies [31]. Based on our experience, this parameter can be used to illustrate some general physiological conditions in plants.

It is known that drought stress causes not only substantial damage to photosynthetic pigments, but also leads to deterioration of thylakoid membranes [38,39]. Thus, a reduction in photosynthetic capacity in plants exposed to drought stress is expected [40], and the decrease in Chl content is a commonly observed phenomenon [41,42]. However, there are also some reports which show an enhanced accumulation of Chl under drought stress [43,44]. Ashraf and Karim [45] suggested that it may be due to the variation in Chl synthesis among the cultivars mediated by the variation in the activities of specific enzymes involved in the biosynthesis of Chl. Chl levels vary with foliage maturity, nutrient levels, environmental conditions, light availability, and also seasonally. Thus, plants continuously regulate their photosynthetic processes in response to the changing environmental conditions on diurnal and seasonal timescales [46]. Our results also showed varied changes in photosynthetic pigment content, through an increase on the 1st day of drought in tested OMA lines, with no alteration in cv. Bingo. We observed a significant decrease in Chl content in lines II and V in the kernel milk maturity phase, which probably is related to the greater sensitivity of these lines to drought associated with accelerated plant aging. On the other hand, line III and cv. Bingo were the only plants characterized by an increase in photosynthetic pigments, both chlorophylls and carotenoids, on the 14th day of drought. Moreover, it is generally known that under drought stress the reduction of Chl*_b_* is greater than that of Chl*_a_* which directly translates into Chl*_a/b_* value [47]. In our study both Chl forms presented quite a similar course of changes within the tested line/cv. Surprisingly, in line I an increase in Chl*_a/b_* occurred despite no significant changes in the content of individual Chl. Similar results were obtained in wheat, where a slight increase in Chl*_a/b_* ratio in drought tolerant cultivars under water deficit conditions was observed [48]. 

Chl absorbed light energy which is then transformed into Chl fluorescence (CF) [49]. In vivo Chl*_a_* fluorescence may be used as a direct indicator of photosynthetic activity giving a valuable insight into the exploitation of the excitation energy by PSII, and indirectly by other protein complexes of the thylakoid membranes [50]. CF delivers the information about the energy absorption, the utilization, and the electron transport in PSII [51]. Therefore, the functional state of the photosynthetic apparatus, based on fluorescence methods, is a useful physiological indicator to study the sensibility of plants to environmental abiotic stress, including drought [52,53,54,55]. Moreover, CF is considered to be a non-invasive tool for the detection of plant photosynthetic performance both under optimal and adverse conditions [33]. CF-modulated parameters are commonly used in the JIP-test. In our study, we assayed CF parameters to characterize the functioning of the photosynthetic apparatus of oat × maize hybrids.

The presented results seem to indicate a different response to drought stress of examined lines, allowing us to divide them, based on the similarity of the value and direction of changes in CF parameters, into two groups: (a) line III and V, in which hardly any significant changes in most parameters were shown, and (b) other hybrid lines and cv. Bingo, characterized by a similar response to applied drought stress (similar course of parameter changes and change of their values i.e., increase/decrease, in drought conditions). Nevertheless, plants of the V line differed from line III with a higher PI and RC/CS_m_, which probably may indicate a more efficient functioning of the photosynthetic apparatus in drought conditions despite the observed decrease in chlorophyll content in drought conditions. Our results revealed some changes in the PI parameter which is sensitive to changes in either antenna properties, trapping efficiency or electron transport beyond QA. The F_v_/F_m_ ratio is the most commonly used CF parameter characterizing the maximum quantum yield of PSII, which in most higher plants under physiological conditions ranges from 0.78 to 0.84. In the presented study, its values were within the range indicated by the literature, regardless of the conditions of plant growth and the date of taken measurements. Furthermore, in oat DH lines after 14 days of drought, F_v_/F_m_ did not differ significantly [55]. In most of the tested OMA lines in drought condition, increased F_v_/F_m_ was not linked to an increase in yield as Liang et al. [56] reported in wheat. There are also some studies where stress did not alter this parameter [57,58]. Its increase observed in several cases in drought conditions seems to be more associated with adaptation to stress than the reflection of damage. Our results are consistent with Antonkiewicz and Rapacz [59] which consider F_v_/F_m_ alteration as a symptom of adaptive or developmental changes. The direction of changes seems to depend on the level of stress because Souza et al. [60] proved that a significant decrease of F_v_/F_m_ occurred only in case of severe drought stress.

Area, ET_0_/CS and RC/CS_0_ showed only slight changes during plant development. An exception was line I, in which these values decreased in drought at panicle ejection phase. Therefore, reduced ET_0_/CS indicates that active reaction centers (RCs) are converted into inactive RCs, reducing the efficiency of trapping and a decline in PSII activity [61]. According to Czyczyło-Mysza et al. [62] higher values of ET_0_/CS, RC/CS_m_, as well as ABS/CS, TR_0_/CS, and PI, indicate better functioning of photosynthetic apparatus. The observed decrease of the ABS/CS ratio under drought stress in our experiment was possibly due to inactivation of some PSII RCs or a decrease in antenna size. In some of the tested OMA lines we observed an increase of RC/CS_m_ which provides information about the number of active reaction centers. In our study most of the tested lines showed higher performance index (PI) values in drought condition which occurred with a simultaneous decrease of energy dissipated from PSII (DI_0_/CS). A similar relationship was observed in the oat DH lines [55]. The relatively constant number of active reaction centers remaining in the tested lines despite drought conditions may probably explain the lack of a significant decrease of energy used for electron transport (ET_0_/CS). 

According to the literature data, OMA lines can be screened for the effects of individual maize chromosomes on photosynthesis in oat [7,9,63]. Since oat is a C3 species and maize is C4, the OMAs may provide a way for the introgression of C4 photosynthesis into oat [7,9,10]. When genes encoding phosphoenolpyruvate carboxylase (PEPC), orthophosphate dikinase (PPdK), pyruvate and the 29-oxoglutarate/malate transporter in maize were expressed in oat, larger bundle sheath cells with increased cell wall lipid deposition were observed in oat leaves [63]. In particular, OMA lines that contained maize chromosomes 6 and 9 were shown to accumulate maize PEPCase and PPdK [7]. Furthermore, both enzymes were active, suggesting that oat PEPC and PPdK regulatory protein can phosphorylate the maize proteins. Moreover, high expression of C4 enzymes in C3 plants may play an important role in improvement of the protection of the photosynthetic apparatus against environmental stress, and hence increase grain yield [64]. However, even in lines with both chromosomes present, photosynthesis was more C3-like than C4 [65]. Despite OMA lines providing insight into certain aspects of C4 regulation they failed to reveal global regulators of the pathway [65], thus the actual function of C4 enzymes in C3 plants still remains unknown [64]. 

### 3.3. Morphology and Yield Components of OMA Lines

Morphological observations of the tested plants revealed some changes within OMA lines. Line I with four maize chromosomes manifested in bushy shoots and poor grain yield. A similar observation was obtained by Riera-Lizarazu et al. [21] who described that plants with three or four maize chromosomes were visibly less vigorous and less viable than haploid plants with no maize chromosomes. In general, hybrids with one or two maize chromosomes appeared more typical and similar to oat plants. The differences in hybrid morphology was also confirmed by Kynast et al. [9]. In their studies, the plants of OMA6 developed necrotic and chlorotic spots on the leaf blades, while OMA9 were characterized by an erratic premature senescence syndrome which led to senescence of single tillers at any stage of development in response to environmental stress (drought and mechanical injury). These phenotypes may be explained by two mechanisms: (i) some ectopic expression of phenotype-linked maize genes can take place under the oat genomic background; (ii) the interaction between oat and maize transcriptome can modify the expression of phenotype-linked oat genes, and the second mechanism seems to be more possible [10]. However, in our experiment such symptoms were not observed. Changes in plant morphology primarily concerned their height, the number of shoots and seeds, and seemed to be more a consequence of applied drought stress rather than the presence of retained maize chromosomes.

## 4. Materials and Methods

### 4.1. Plant Material and Growth Conditions

The objects of research in this study were oat plants (*Avena sativa* L.) cv. Bingo and 5 selected OMA lines, DC 06011-6 × POB 722 (I), STH 9511 × Bingo (II), STH 8-50 × Canyon (III), STH 9110 × Contender (IV) and STH 93-61 × DC 2112/07 (V), which differed in the number of maize chromosomes added to the oat genome. The 10 plants per line/cultivar were grown in pots under an open-air vegetation tunnel protected from rain by gardening foil during the summer–autumn period (July–September). Plants were grown in conditions close to natural daylight and air temperature. The average temperatures recorded in these three months were 25 °C (32–19 °C), 20 °C (30–10 °C) and 15 °C (25–5 °C), while the air humidity was as follows: 70%, 75%, 80%. Oat seeds of cv. Bingo and the OMA lines were planted individually into 3 dm^3^ pots filled with soil composed of horticultural soil (loamy soil (85%) and loam (15%)) and sand (1:1 *v*/*v*). Drought stress was caused by withholding the watering of the soil as described by Marcińska et al. [55] when the plants reached the seedling phase (3–4 leaves). Half plants of each line/cultivar were subjected to drought. The degree of soil moisture was determined by the weighing pots and set at 70% of field water capacity (FWC) for control (C) and 25% FWC for drought (D) conditions. During the experiment the water content in soil was controlled gravimetrically, including the mass of plants growing in the pots, and by using a moisture meter MO750 (Extech Instruments Corporation, USA). Drought treatment was continued for 14 days. After then, all plants were watered to 70% FWC to allow for further analysis of the yield components. 

The experiments was conducted at The *Franciszek Górski* Institute of Plant Physiology Polish Academy of Sciences (50°47′ N, 19°50′ E), Kraków, Poland. 

### 4.2. Identification of Oat × Maize Hybrids

DNA extraction (Genomic Mini AX Plant Kit, A&A Biotechnology, Gdynia, Poland) and PCR analyses (2720 Thermal Cycler; Applied Biosystems, Foster City, CA, USA) were performed according to Skrzypek et al. [66]. Two Grande 1F (5′-AAA GAC CTC ACG AAA GGC CCA AGG-3′) and Grande1R (5′-AAA TGG TTC ATG CCG ATT GCA CG-3′) primers (GenBank accession number X97604; [2]) were used for the PCR reaction, which in successive cycles enabled the amplification of the 500 bp retrotransposon region of Grande-1 and detection of the presence of maize chromatin in oat plants. The obtained products were separated in 1.5% agarose gel with ethidium bromide (Sigma-Aldrich, St. Louis, MO, USA) in TBE buffer, under 90 V for 90 min. DNA markers of 100 bp to 1000 bp and concentration of 0.5 mg/mL (GeneRuler 100bp; Fermentas, Waltham, MA, USA) were used to estimate the length of PCR products. The image of electrophoretic separation was archived using the Imagemaster VDS gel reader (Amersham, Pharmacia Biotech, Piscataway, NJ, USA) and the Liscap Capture Application ver. 1.0. Electrophoretic gel analysis was performed using GelScan ver. 1.45 (Kucharczyk Electrophoretic Techniques, Warsaw, Poland). The plants identified as having a Grande-1 retrotransposon fragment were used for genomic in situ hybridization (GISH).

### 4.3. Mitotic Chromosome Preparations

The chromosome preparations were made using the methodology described by Jenkins and Hasterok [67]. The root tips of 3-day-old seedlings were excised, incubated in ice-cold water for 24 h, and fixed in a mixture of methanol and glacial acetic acid at a 3:1 ratio. The material was stored in −20 °C. The root tips were washed in citrate buffer (pH 4.8) for 15 min, and then digested in an enzyme mixture containing 20% pectinase (Sigma), 1% cellulase (Calbiochem, San Diego, CA, USA), and 1% cellulase ‘Onozuka R-10′ (Serva, Heidelberg, Germany) in citrate buffer at 37 °C for 2 h. After digestion, the meristems were dissected from the root tips, placed in a drop of acetic acid on a microscopic slide, covered with a coverslip, and gently squashed. Good quality slides were frozen on dry ice. After removing the coverslips, the slides were air dried and stored at 4 °C until used.

### 4.4. Probe Labelling and in Situ Hybridization

For GISH experiments, maize total genomic DNA (gDNA) was extracted from young leaves using standard C-TAB procedure and labelled with digoxygenin-11-dUTP (Roche, Basel, Switzerland) by nick-translation method according to the manufacturer instructions. In some experiments, 25S rDNA sequence labelled with tetramethylrhodamine-5-dUTP by nick translation was used as an additional chromosome marker. The GISH procedure followed the protocol described in Skrzypek et al. [66] with modifications. Briefly, the slides were pre-treated with RNase (100 mg/mL) in 2 × saline sodium citrate (SSC) buffer for 1 h, washed several times in 2 × SSC, dehydrated in ethanol series (70%, 90%, 100%), and air dried. The labelled probe(s) DNA (~500 ng/slide) was precipitated, dried and dissolved in hybridization mixture which consisted of 50% formamide, 10% dextran sulfate, 20 × SSC, and water. Chromosome preparations and predenatured (10 min at 75 °C) hybridization mixtures were denatured together for 4.5 min at 75 °C and allowed to hybridize in a humid chamber for about 42–48 h at 37 °C. Post-hybridization washes were performed in 10% formamide in 0.1 × SSC at 42 °C (79% stringency). The hybridization signals were detected by antidigoxigenin fluorescein-conjugated antibodies (Roche, Basel, Switzerland) or, in the case of tetramethyl-rhodamine-5-dUTP, visualized directly. The chromosomes were mounted and counterstained in VectaShield Antifade (Vector Laboratories, Burlingame, CA, USA) containing 2.5 mg/mL DAPI (Serva, Heidelberg, Germany). All microphotographs were acquired using fluorescence microscope Axio Imager Z2 equipped with monochromatic camera AxioCamMRm (ZEISS, Oberkochen, Germany). The acquired images were digitally processed and superimposed using ZEN blue program (ZEISS, Oberkochen, Germany) and Photoshop CS3 (Adobe, San Jose, CA, USA).

### 4.5. Maize Chromosome Identification

The total genomic DNA of OMA lines, oat cv. Bingo, and maize cv. Waza was extracted from ca. 0.8 g of fresh tissue frozen with liquid N_2_. DNA isolation was performed using DNeasy Plant Mini Kit (Qiagen, Hilden, Germany). The concentration of DNA was measured at 260 nm by NanoDrop 2000c (Thermo Scientific, Waltham, MA, USA) and its quality was checked on agarose gel. The added maize chromosomes in the tested OMA lines were identified with specific for maize SSR markers chosen from the Maize Genome Database (https://www.maizegdb.org) and described by [68]. 

### 4.6. Leaf Relative Water Content 

Leaf relative water content (LRWC) was determined on the 14th day of drought according to Ober et al. [69] using the following formula: LRWC (%) = (Wf − Wd)/(Wt − Wd) × 100, where Wf, Wd and Wt represent fresh mass, dry mass and turgid mass, respectively. Samples were collected from the second fully developed leaf from each independent plant.

### 4.7. Chlorophyll Concentration in Leaves

The chlorophyll concentration indicator in SPAD units was determined by a photometric method using a SPAD 502 device (Konica Minolta Sensing Europe B.V., Warrington, UK) based on the leaf greenness index. By measuring the wavelength of 650 nm, the amount of light absorbed by the chlorophyll was determined. In addition, light was measured at a wavelength of 940 nm which is absorbed by the rest of the structure. These values were used to correct the result calculated by the microprocessor and given in contractual units, called SPAD readings (with maximum at 60 units). SPAD readings are directly proportional to the chlorophyll content. The chlorophyll concentration indicator in SPAD units was measured on four terms: on the 1st, 14th day of drought, panicle ejection phase (PE), and kernel milk maturity phase (MM). The measurements were taken in the middle part of the second or third fully developed leaf (counting from the youngest leaf) from each independent plant.

### 4.8. Photosynthetic Pigments 

The content of photosynthetic pigments was determined on the 1st, 14th day of drought, panicle ejection phase, and kernel milk maturity phase by a modified spectrophotometric method according to Lichtenthaler and Wellburn [70]. Leaf samples (5 mg) were homogenized in 80% ethanol. The homogenate was centrifuged at 1000 g for 5 min at 4 °C (Eppendorf Centrifuge 5702 R centrifuge, Germany). Then, the supernatant was stored at 4 °C in the dark until absorbance was measured. The absorbance value was measured using a Synergy 2 spectrophotometer, BioTek (Winooski, VT, USA), at wavelengths λ = 470 nm, 648 nm and 664 nm. The contents of chlorophyll *a* and *b* and carotenoids were calculated using the following formula:

Chl*_a_* = 12.7A_664_ − 2.7A_648_

Chl*_b_* = 22.9A_648_ − 4.7A_664_

Car = (1000A_470_ − 2.13Chl*_a_* − 97.64Chl*_b_*)/209

where Chl*_a_* = chlorophyll *a*, Chl*_b_* = chlorophyll *b*, Car = carotenoids, A_664_ = absorbance at 664 nm, A_648_ = absorbance at 648 nm; A_470_ = absorbance at 470 nm. The concentrations was expressed in micrograms of a given pigment in 1 mL of extract.

### 4.9. Chlorophyll A Fluorescence Parameters

The measurements of the chlorophyll *a* fluorescence kinetics parameters were conducted using a Handy PEA fluorometer (Hansatech, Kings Lynn, UK). The measurements were carried out on the same leaves on which the chlorophyll concentration indicator in SPAD unit had been measured previously on the four terms mentioned above. The leaves’ fragments were shaded with clips for about 20 min and then the measurement was taken. It was initiated by illuminating the leaf fragment with saturated light (intensity of 90 units = 5400 μmol (quantum) m^2^s^−1^, illumination time 0.9 s) to determine the maximum fluorescence (F_m_) and calculate the maximum photochemical potential (F_v_/F_m_). Then, actinic light was switched on automatically (25 units = 1500 μmol (quantum) m^−2^s^−1^) for a period of 270 s. When it was switched off, the leaf light was emitted by a diode emitting light in the far red range of about 15 W m^−2^. On the basis of chlorophyll *a* fluorescence measurements, the following parameters were calculated and analyzed: F_v_/F_m_—maximum photochemical efficiency of PSII; Area—area over the chlorophyll *a* fluorescence induction curve; PI—overall performance index of PSII photochemistry; ABS/CS—light energy absorption; TR_0_/CS—excitation energy trapped in PSII reaction centers; ET_0_/CS—energy used for electron transport; DI_0_/CS—energy dissipated from PSII; RC/CS_0_—number of active reaction centers in the excited leaf fragment; RC/CS_m_—number of active reaction centers in the relaxation state of leaf fragment.

### 4.10. Yield Component Analysis

To estimate the impact of drought on plants, morphological observations were carried out and selected components of yield (plant height, number of side shoots, number of grains, mass of grains) were assessed.

### 4.11. Statistical Data Analysis

The results presented in figures constitute the mean values ± SE based on 5 plants as replicates per treatment. One-way and multivariate analysis of variance and Duncan’s multiple range test at the 0.05 probability level were used to determine the significance of differences between lines/cultivars, which were marked with different letters. Student’s t-test at the 0.05 probability level was also used to compare the average values for each line/cultivar. Principal component analysis (PCA) was done and presented as biplot. Data were analyzed using STATISTICA v. 13.1 software (TIBCO Software Inc., Palo Alto, CA, USA).

## 5. Conclusions

Our studies have revealed the complexity of interspecific hybrid functioning and some of the consequences of preserving alien chromosome(s). Obtained results of CF parameters and pigment content can indicate that OMA lines may predominate oat cultivar in terms of photosynthetic apparatus functioning both in optimal and adverse conditions. We postulate that the functioning of the photosynthetic apparatus in OMA lines depends not only on the number of preserved maize chromosomes but on their interaction with a given oat genome. Although the whole oat genome sequence is currently unavailable, additional studies of the effects of maize chromosomes on oat transcriptome in OMA lines should be continued to explore the mechanism underlying their adjustment to adverse environmental conditions. Possibly, generating crop hybrids may contribute to solving the problem of further production level enhancement, which currently is rather limited. As ways to increase the yield potential, an improvement of photosynthesis and ascension of photosynthetic utilization are mentioned [71].

## Figures and Tables

**Table 1:** Characteristics of the plant material used in the study.

Genotype/Cultivar	Origin	No. of Maize Chromosome/Chromatin Added to Oat Genome	Chromosome ID
I	DC 06011-6 × POB 722	4	1, 2
II	STH 9511 × Bingo	2	2
III	STH 8-50 × Canyon	1	3
IV	STH 9110 × Contender	2	5
V	STH 93-61 × DC 2112/07	Chromatin	6
Bingo	-	-	-

**Table 2:** *F*-statistic from one-way analysis of variance of the traits measured in control oat plants. SPAD—total chlorophyll content; Chl*_a_*—chlorophyll *a*; Chl*_b—_*chlorophyll *b*; Chl*_a/b—_*chlorophyll *a/b* ratio; Car—carotenoids; F_v_/F_m_—maximum photochemical efficiency of PSII; Area—area over the chlorophyll *a* fluorescence induction curve; PI—overall performance index of PSII photochemistry; ABS/CS—light energy absorption; TR_0_/CS—excitation energy trapped in PSII reaction centers; ET_0_/CS—energy used for electron transport; DI_0_/CS—energy dissipated from PSII; RC/CS_0_—number of active reaction centers in the excited leaf fragment; RC/CS_m_—number of active reaction centers in the relaxation state of leaf fragment.

Source of Variance	Trait	*F*
Total chlorophyll content	SPAD	18.762 ***
Photosynthetic pigment content	Chl*_a_*	10.692 ***
	Chl*_b_*	13.243 ***
	Chl*_a/b_*	6.120 ***
	Car	21.941 ***
Chlorophyll *a* fluorescence parameters	F_v_/F_m_	2.250 ^ns^
	Area	4.226 **
	PI	4.786 **
	ABS/CS	3.307 *
	TR_0_/CS	5.004 ***
	ET_0_/CS	7.308 ***
	DI_0_/CS	1.800 ^ns^
	RC/CS_0_	13.360 ***
	RC/CS_m_	5.979 *

^ns^ not significant. *, **, *** significant at *p* ≤ 0.05, 0.01, 0.001, respectively.

**Table 3:** *F*-statistic from three-way analysis of variance for photosynthetic pigments of studied genotypes (OMA lines and cv. Bingo).

Trait/Interaction	Degrees of Freedom	LRWC	SPAD	Chl*_a_*	Chl*_b_*	Chl*_a/b_*	Car
Genotype	5 (k − 1)	1.837 ^ns^	54.415 ***	9.746 ***	8.389 ***	19.605 ***	16.285 ***
Treatment	1 (t − 1)	6.568 *	28.666 ***	9.892 **	1.549 ^ns^	50.340 ***	0.073 ^ns^
Term	3 (n − 1)	-	539.706 ***	46.231 ***	71.463 ***	1.921 ^ns^	52.673 ***
Genotype × Treatment	5 (k − 1) × (t − 1)	0.898 ^ns^	1.375 ^ns^	2.158 ^ns^	1.537 ^ns^	3.595 ***	0.832 ^ns^
Genotype × Term	5 (k − 1) × (n − 1)	-	9.252 ***	4.751 ***	3.540 **	6.135 ***	5.587 ***
Treatment × Term	1 (t − 1) × (n − 1)	-	5.803 ***	33.310 ***	28.344 ***	34.415 ***	36.859 ***
Genotype × Treatment × Term	5 (k − 1) × (t − 1) × (n − 1)	-	2.331 **	6.411 ***	7.329 ***	3.105 ***	4.861 ***

* *p* ≤ 0.05, ** *p* ≤ 0.01, *** *p* ≤ 0.001, ns—not significant, k—number of lines, t—treatment (control and drought), n—term (1st day of drought, 14th day of drought, panicle ejection phase, kernel milk maturity phase).

**Table 4:** *F*-statistic from three-way analysis of variance for chlorophyll *a* fluorescence parameters of studied genotypes (OMA lines and cv. Bingo).

Trait/Interaction	F_v_/F_m_	Area	PI	ABS/CS	TR_0_/CS	ET_0_/CS	DI_0_/CS	RC/CS_0_	RC/CS_m_
Genotype	12.326 ***	29.475 ***	6.750 ***	7.438 ***	6.853 ***	10.090 ***	8.317 ***	15.872 ***	26.191 ***
Treatment	51.637 ***	5.143 *	86.553 ***	50.093 ***	52.487 ***	5.193 *	44.822 ***	0.581 ^ns^	71.659 ***
Term	11.938 ***	111.585 ***	65.181 ***	26.533 ***	49.481 ***	115.331 ***	12.478 ***	335.934 ***	150.815 ***
Genotype × Treatment	5.798 ***	6.012 ***	4.742 ***	6.833 ***	7.777 ***	4.684 ***	6.085 ***	3.817 ***	5.412 ***
Genotype × Term	2.841 ***	6.401 ***	2.896 ***	2.856 ***	4.181 ***	4.769 ***	2.118 **	5.566 ***	5.929 ***
Treatment × Term	17.652 ***	2.904 *	16.365 ***	22.345 ***	29.979 ***	22.270 ***	15.271 ***	3.851 **	13.236 ***
Genotype × Treatment × Term	2.381 **	2.876 ***	2.408 **	3.062 ***	3.964 ***	3.319 ***	2.347 **	4.095 ***	2.397 **

* *p* ≤ 0.05, ** *p* ≤ 0.01, *** *p* ≤ 0.001, ns—not significant, degrees of freedom see Table 3.

**Table 5:** *F*-statistic from two-way analysis of variance for selected traits of studied genotypes (OMA lines and cv. Bingo).

Source of Variation	Degrees of Freedom	Height of Main Shoot	Panicle Length	Number of Shoots	Weight of Shoots/Plant	Number of Seeds/Plant	Weight of Seeds/Plant
Genotype	5 (k − 1)	34.699 ***	21.807 ***	14.157 ***	39.379 ***	3.030 *	13.536 ***
Treatment	1 (t − 1)	49.988 ***	31.642 ***	4.823 *	26.811 ***	18.039 ***	0.121 ^ns^
Genotype × Treatment	5 (k − 1) × (t − 1)	7.548 ***	2.603 ***	0.715 ^ns^	8.836 ***	0.397 ^ns^	2.831 **

* *p* ≤ 0.05, ** *p* ≤ 0.01, *** *p* ≤ 0.001, ns—not significant, k—number of genotypes, t—treatment (control and drought).

## Abbreviations

ABS/CS	Light Energy Absorption
Area	Area over the chlorophyll *a* fluorescence induction curve
Car	Carotenoids
CF	Chlorophyll Fluorescence
Chl	Chlorophyll
DI_0_/CS	Energy dissipated from PSII
DM	Dry Mass
ET_0_/CS	Energy used for electron transport
FM	Fresh Mass
F_v_/F_m_	Maximum photochemical efficiency of PSII
FWC	Field Water Capacity
LRWC	Leaf Relative Water Content
OMA	Oat × maize chromosome addition line
PCA	Principal Components Analysis
PC	Principal Component
PI	Overall performance index of PSII photochemistry
RC/CS_m_	Number of active reaction centers in the relaxation state of leaf fragment
RC/CS_0_	Number of active reaction centers in the excited leaf fragment
SPAD	Soil Plant Analysis Development
TR_0_/CS	Excitation energy trapped in PSII reaction centers
